# Metal complexes as a promising source for new antibiotics†
†Electronic supplementary information (ESI) available. See DOI: 10.1039/c9sc06460e


**DOI:** 10.1039/c9sc06460e

**Published:** 2020-02-12

**Authors:** Angelo Frei, Johannes Zuegg, Alysha G. Elliott, Murray Baker, Stefan Braese, Christopher Brown, Feng Chen, Christopher G. Dowson, Gilles Dujardin, Nicole Jung, A. Paden King, Ahmed M. Mansour, Massimiliano Massi, John Moat, Heba A. Mohamed, Anna K. Renfrew, Peter J. Rutledge, Peter J. Sadler, Matthew H. Todd, Charlotte E. Willans, Justin J. Wilson, Matthew A. Cooper, Mark A. T. Blaskovich

**Affiliations:** a Centre for Superbug Solutions , Institute for Molecular Bioscience , The University of Queensland , St. Lucia , Queensland 4072 , Australia . Email: angelo.frei.ch@gmail.com ; Email: m.blaskovich@uq.edu.au; b School of Molecular Sciences , The University of Western Australia , Stirling Highway , 6009 Perth , Australia; c Institute of Organic Chemistry , Karlsruhe Institute of Technology (KIT) , Fritz-Haber-Weg 6 , 76131 Karlsruhe , Germany; d Institute of Biological and Chemical Systems – Functional Molecular Systems (IBCS-FMS) , Karlsruhe Institute of Technology (KIT) , Hermann-von-Helmholtz-Platz 1 , D-76344 Eggenstein-Leopoldshafen , Germany; e School of Medical Sciences (Discipline of Pharmacology) , University of Sydney , Australia; f Department of Chemistry , University of Warwick , Gibbet Hill Road , Coventry CV4 7AL , UK; g Antimicrobial Screening Facility , School of Life Sciences , University of Warwick , Gibbet Hill Road , Coventry CV4 7AL , UK; h Institute of Molecules and Matter of Le Mans (IMMM) , UMR 6283 CNRS , Le Mans Université , France; i Department of Chemistry and Chemical Biology , Cornell University , Ithaca , NY 14853 , USA; j Chemistry Department , Faculty of Science , Cairo University , Egypt; k School of Molecular and Life Sciences – Curtin Institute for Functional Materials and Interfaces , Curtin University , Kent Street , 6102 Bentley WA , Australia; l School of Chemistry , University of Leeds , Woodhouse Lane , Leeds LS2 9JT , UK; m School of Chemistry , The University of Sydney , Sydney , NSW 2006 , Australia; n School of Pharmacy , University College London , London , WC1N 1AX , UK

## Abstract

There is a dire need for new antimicrobial compounds to combat the growing threat of widespread antibiotic resistance. With a currently very scarce drug pipeline, consisting mostly of derivatives of known antibiotics, new classes of antibiotics are urgently required. Metal complexes are currently in clinical development for the treatment of cancer, malaria and neurodegenerative diseases. However, only little attention has been paid to their application as potential antimicrobial compounds. We report the evaluation of 906 metal-containing compounds that have been screened by the Community for Open Antimicrobial Drug Discovery (CO-ADD) for antimicrobial activity. Metal-bearing compounds display a significantly higher hit-rate (9.9%) when compared to the purely organic molecules (0.87%) in the CO-ADD database. Out of 906 compounds, 88 show activity against at least one of the tested strains, including fungi, while not displaying any cytotoxicity against mammalian cell lines or haemolytic properties. Herein, we highlight the structures of the 30 compounds with activity against Gram-positive and/or Gram-negative bacteria containing Mn, Co, Zn, Ru, Ag, Eu, Ir and Pt, with activities down to the nanomolar range against methicillin resistant *S. aureus* (MRSA). 23 of these complexes have not been reported for their antimicrobial properties before. This work reveals the vast diversity that metal-containing compounds can bring to antimicrobial research. It is important to raise awareness of these types of compounds for the design of truly novel antibiotics with potential for combatting antimicrobial resistance.

## Abstract 2

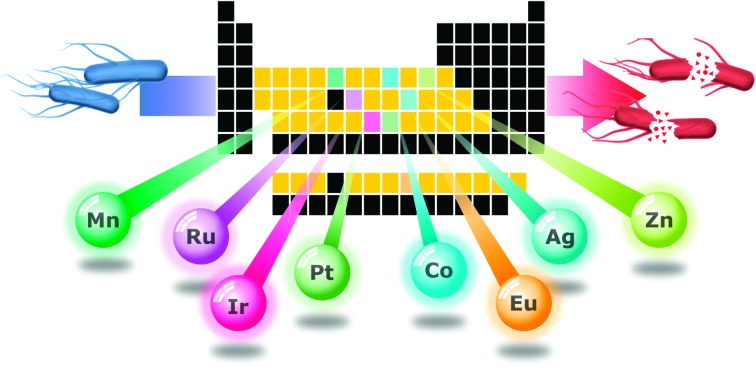
There is a dire need for new compounds to combat antibiotic resistance: metal complexes might provide the solution. 906 metal complexes were evaluated against dangerous ESKAPE pathogens and found to have a higher hit-rate than organic molecules.

## Introduction

The scientific community is struggling to keep up with the pace at which bacterial infections are evading antibiotics through the development of multidrug resistance. As of July 2019, there were 42 antibiotic drug candidates in clinical trials.^1^ While this may sound encouraging, the context is important: In 2018 there were over 1100 medicines and vaccines in clinical trials as cancer treatments.^80^ Furthermore, almost 75% of the antimicrobials under clinical development are simply derivatives of already known and used antibiotics, meaning that they will likely be prone to existing resistance mechanisms. To make things worse, only one of the remaining 11 entirely new compounds is effective against the notoriously more resilient Gram-negative strains.^1^ Commercial development of new antibiotics is unlikely to refill the antibiotic pipeline in the near future, with the number of pharmaceutical companies actively researching new antibiotics still shrinking every year due to the unfavorable return on investment in the field.^2^,^3^

A recurrent trend in the antibiotic chemical landscape is that antibacterial compounds are rarely “drug-like”, *i.e.* they do not conform to the physicochemical and reactive functionality guidelines developed to direct the discovery of new orally available therapies. This means that many potential new antibiotics have already been excluded from synthetic libraries that are designed with these common drug-design axioms in mind, particularly those developed within the pharmaceutical industry (*e.g.* following Lipinski's Rule of Five^4^). To circumvent this inherent medicinal chemistry bias, our research group has embarked on a quest to tap into the millions of diverse compounds prepared in academic chemistry laboratories across the world, new molecules with a range of shapes and sizes that have been synthesized for methodology development or for other biological applications, and which are often simply sitting on a shelf somewhere, unused. In the last four years the Community for Open Antimicrobial Drug Discovery (CO-ADD) has offered scientists worldwide access to a simple and free screening service where any submitted compound could be evaluated against key bacterial and fungal pathogens.^5^,^6^ By leaving all IP rights in the hands of the originators and employing a widespread campaign, marketing our service, we have received and screened over 295 000 compounds from 280 academic groups from 46 countries. A substantial proportion of compounds submitted to CO-ADD fall within the accepted definitions of “drug-likeness”, demonstrating the extent to which these rules have permeated even academic research efforts. Fortunately, we have also received a considerable number of compounds that lie outside such conventions, with, for example, low lipophilicity (log *D* < 2) and larger molecular weight (*M*_W_ > 500). In addition, a considerable number of metal-containing compounds were found amongst the submitted compounds, which are the focus of this report.

A common denominator of lead structures in the pharmaceutical industry and most compounds developed by medicinal chemists across the globe is that they are almost exclusively purely organic compounds. At first sight, this might seem intuitive as metals and their complexes are mostly known for their application as materials or catalysts and are often associated with toxicity. However, metal-based coordination complexes have played a crucial role in medicine throughout history, including the use of arsenic for the first effective treatment of syphilis (Salvarsan), mercury in the topical antiseptic mercurochrome or the vaccine preservative thiomersal, and gold in the treatment of rheumatoid arthritis (Auranofin).^7^,^8^ Metal complexes became a cornerstone of medicinal chemistry with the approval of the chemotherapeutic platinum-based drug Cisplatin (1978), which is still used in a majority of all cancer treatments today.^9^ In the last two decades, several more titanium-, iron-, ruthenium-, gallium-, palladium-, silver-, gold-, bismuth-, and copper-based metal complexes have reached human clinical trials as treatments for cancer, malaria and neurodegenerative diseases.^10^–^13^,^81^,^82^ A palladium-based photodynamic therapy agent was approved in 2019 for the treatment of prostate cancer by the European Medicines Agency (EMA).^83^ Many more elements are actively being investigated for a range of medical applications.^14^,^15^

However, only a few studies have focused on antibacterial applications. Bismuth- and silver-based antimicrobials are the only elements used in some clinical interventions. Bismuth is co-administered in combination with other antibiotics for the treatment of *Helicobacter pylori* infections.^16^–^18^ Xeroform, namely tribromophenatebismuth(iii), is used as an antimicrobial in wound dressing applications.^19^ A bismuth-thiol compound (pravibismane) just completed a phase 1b clinical trial for the treatment of infected diabetic foot ulcers.^20^ Silver is also used in a variety of forms for the treatment of wounds and management of infections.^16^ Silver sulphadiazine is applied either as a cream or aqueous solution for the topical treatment of some burn wounds. However, the effectiveness of these silver compounds has been questioned in the last years. In recent trials, topically applied silver-based compounds performed only equal or worse than non-silver based treatments.^21^–^23^

Despite these few, rather unspecific metal-based treatments, the inorganic chemistry space is still largely ignored for antimicrobial applications. This is unfortunate because the tremendous variety of three-dimensional structural scaffolds available through metal coordination chemistry is an ideal starting point for the exploration of novel chemical space for new antibiotic compounds and provides a ready “escape from flatland”.^24^,^25^ This argument has recently been validated through seminal work by Morrison *et al.* The authors show that while the majority of organic molecules have simple one- or two-dimensional shapes, metal complexes can easily access hitherto underexplored three-dimensional chemical space. A small library of 71 ‘metallofragments’ was assembled and shown to cover a significantly larger portion of the available geometrical space with 77% of the compounds possessing a three-dimensional shape (compared to 25% of a 18 435 molecule library of organic fragments).^26^

In addition, metal complexes have access to unique modes of action: ligand exchange or release, ROS generation, redox activation and catalytic generation of toxic species or depletion of essential substrates. Such mechanisms are difficult if not impossible to replicate with purely organic compounds.^27^,^28^

We scanned the CO-ADD database for metal-containing compounds and found close to 1000 structures that had been submitted and evaluated over the last four years. Herein we report the analysis of the antibacterial and antifungal activity of what is to date the largest dataset of metal complexes tested for antimicrobial activity. We show that metal-based compounds display a significantly higher hit-rate against critical ESKAPE pathogens and fungi when compared to the solely organic compounds within the CO-ADD collection. While the data are still too sparse for any specific structure–activity relationship evaluation, we believe this study can serve as an important roadmap, highlighting which metals and ligands have proven promising and are therefore worthy of further study and, conversely, which ones have not yet been explored. We hope that through this work we can encourage more researchers worldwide to submit their metal compounds for screening in order to expand this open library and to gather more detailed data.

## Results and discussion

### Scope

**Fig. 1:**
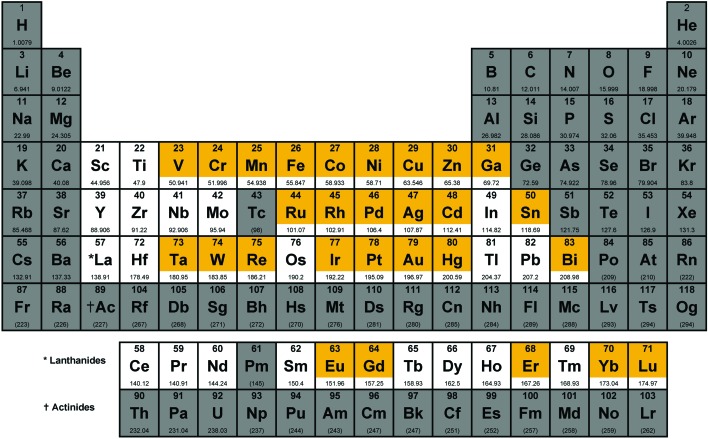
Periodic table highlighting the elements classified as metals for the purpose of this study (white) and the subset of these elements that are contained in compounds that have been submitted to CO-ADD to date (orange). Figure adapted with permission from Nessa Carson (www.supersciencegrl.co.uk).

**Fig. 2:**
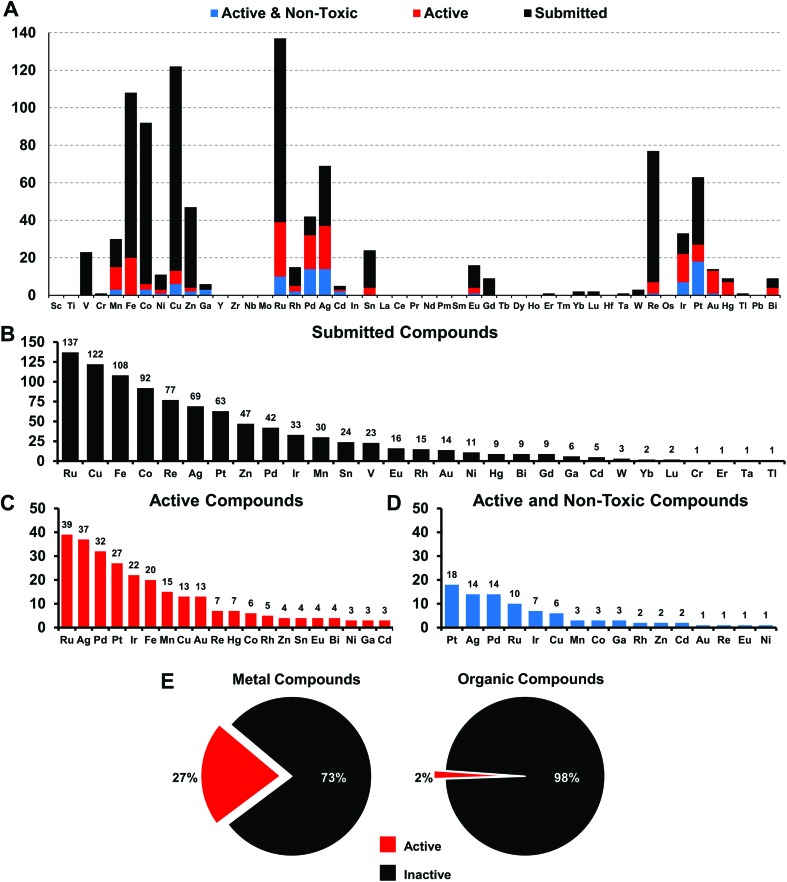
(A) Elemental distribution of all 906 metal-containing compounds submitted to CO-ADD (black); 246/906 submitted metal-containing compounds with at least one MIC lower or equal to 16 μg mL^–1^ or 10 μM (red) against the tested organisms; 88/246 active metal-containing compounds with no cytotoxicity or hemolytic activity at the highest concentration tested (blue). (B) Metal frequency amongst the 906 metal-containing compounds submitted to CO-ADD. (C) Metal frequency amongst the 246 metal complexes that possess some activity against the tested organisms. (D). Metal frequency amongst the 88 compounds that are active as well as ‘non-toxic’ (see text for definition). (E) Percentage of submitted metal-containing compounds with antimicrobial activity, compared to the overall hit rate for organic compounds within the CO-ADD collection.

We have defined the scope of the term metal complex to include all d-block elements and the lanthanides, as well as the post-transition metals gallium, indium, tin, thallium, lead and bismuth, but have excluded the actinides and the rare/radioactive d-elements with atomic number >100, as well as the radioactive elements technetium and promethium, leaving 49 elements. A total of 906 individual compounds containing 29 of these 49 possible metal elements (Fig. 1) have been evaluated by CO-ADD (as of July 2019). These compounds have been submitted by 47 research groups from 17 different countries across the world. Fig. 2 shows a detailed elemental distribution of the compounds submitted (black bars). Amongst the submissions, 63 compounds contained two metal centers.

The more abundant first-row d-block elements represent almost 50% of all compounds. However, it is the second-row transition metal ruthenium that has the highest number of submissions. This is likely a consequence of the trend of ruthenium-based compounds for anticancer applications in the last years, fueled by the advancement into clinical trials of NAMIA-A, KP1019 and very recently TLD1433, which has led to many inorganic medicinal chemistry groups investigating these types of compounds.^12^

Compounds are submitted to CO-ADD as dry powders, confirmed to be at >95% purity by the collaborators, and then tested as received. Characterization of the complexes is done by the submitting research group. No quality control check of purity is performed by CO-ADD due to the volume of compounds received; false positives are therefore possible and promising compounds should be checked thoroughly before further development. The antimicrobial assays are done in duplicates and the data added to the database only if consistent results are obtained between runs. Compounds that are submitted to CO-ADD first undergo a primary screening where they are tested at a fixed concentration (usually 32 μg mL^–1^ or 20 μM, depending on the provided stock solution) against key bacterial (*Escherichia coli*, *Klebsiella pneumoniae*, *Acinetobacter baumannii*, *Pseudomonas aeruginosa*, methicillin resistant *Staphylococcus aureus* (MRSA)) and fungal (*Cryptococcus neoformans* (yeast) and *Candida albicans*) pathogens. If the compound shows any significant inhibition at this concentration, a follow-up hit confirmation is triggered, where the activity is measured by means of a dose–response assay against the same strains. We filtered the 906 metal complex entries to identify compounds with at least a minimal amount of activity, defined as a minimum inhibitory concentration (MIC) value of equal to or lower than 16 μg mL^–1^ (or 10 μM) against at least one organism. A total of 246 compounds qualified as active under these criteria, representing a hit-rate of 27% over the entire set of metal compounds. In comparison, the overall hit-rate for CO-ADD is 1.6% (4620 actives out of 287 385 screened, excluding the metal compounds). While this substantially higher hit-rate is extraordinary, it is conceivable that many metal complexes simply possess general toxicity that is not specific to bacteria but also affects human cells. Given that a significant subset of these compounds was made with anticancer applications in mind, where cytotoxic properties are desirable, this is a concern. To address this issue, toxicity was assessed by measuring mammalian cell viability (HEK293 human embryonic kidney cells) and haemolytic activity against human red blood cells. Non-toxic compounds were defined as compounds with HEK293 CC_50_ > 32 μg mL^–1^ or >20 μM and haemolytic HC_10_ > 32 μg mL^–1^ or >20 μM (HC_10_ is the concentration causing 10% haemolysis). Removing all hits that displayed toxicity and/or haemolysis left 88 metal-containing compounds, a hit-rate of 9.9% for non-cytotoxic hits, still substantially enhanced (>10-fold higher) compared to 0.87% for the general CO-ADD library. Of note, the percentage of toxic and/or haemolytic compounds was found to be nearly identical when comparing the metal compounds with the rest of the CO-ADD library (64.2% *vs.* 64.5%). Overall, this analysis indicates that a higher degree of general toxicity can be ruled out as the cause for the extraordinarily high hit-rate for active metal-bearing compounds compared to organic molecules.

**Fig. 3:**
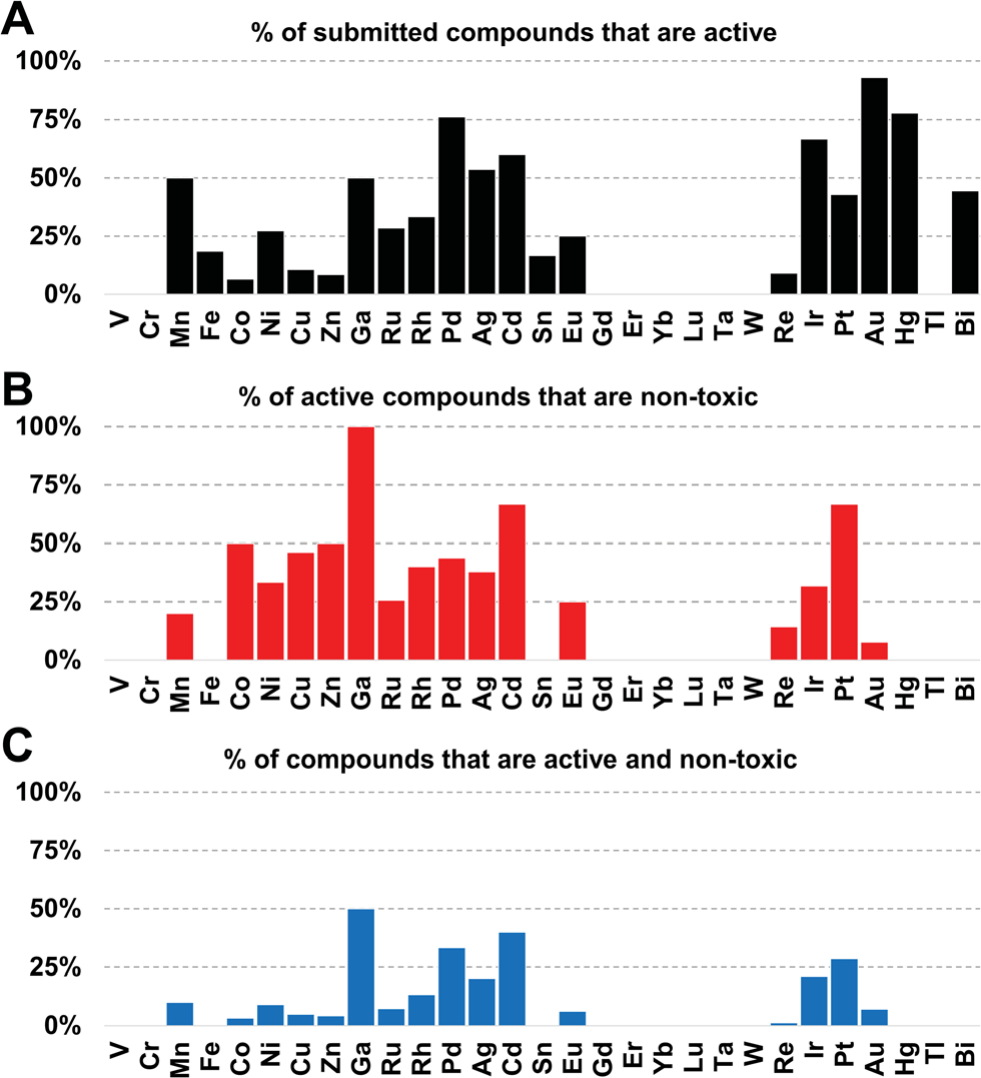
(A) Percentage of submitted compounds that were found to be active, classified by element. (B) Percentage of submitted compounds that were found to be active and ‘non-toxic’, classified by compounds per element that are also non-toxic. (C) Overall success-rate of compounds, classified by element.

Amongst the metal complexes, ruthenium was the most frequent element found in active ‘non-toxic’ compounds, followed by silver, palladium and iridium. Copper, iron, cobalt and zinc stand out as ‘underperformers’, with fewer than 20% of the submitted complexes showing any activity at all (Fig. 3). In general, first-row transition metal ions undergo ligand substitution more rapidly than the second and third row elements. This could indicate that these compounds react more quickly with components in the medium and are not able to enter the bacterial cells. Another possibility is that the bacteria are able to efflux essential metals more easily as their biochemistry is familiar to them.

**Fig. 4:**
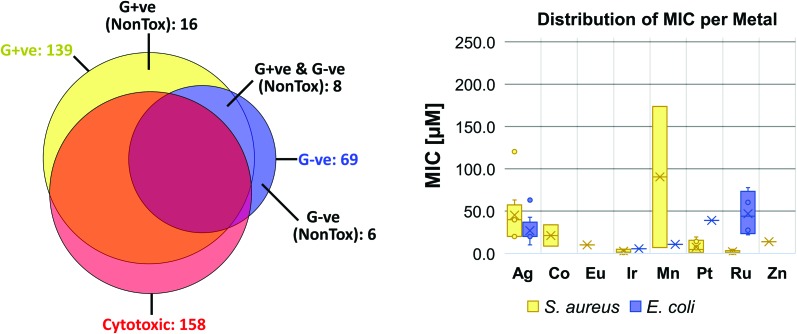
Activity distribution of metal complexes between the different classes of bacteria (left) and as distribution of the MIC for *S. aureus* (MRSA, ATCC 43300) and *E. coli* (ATCC 25922) shown as box plot (first and third quartile) with median (line), mean (cross) and outliers (dots).

When these compounds are further broken down according to their activity profile, 58 of them were exclusively antifungal, while the other 30 were active against at least one bacterial strain (Fig. 4). As antibacterial agents, most entries showed the best activity against Gram-positive MRSA, as is generally true for the entire set of CO-ADD screened compounds. Finally, only 14 compounds showed any activity (MIC ≤ 32 μg mL^–1^) against the Gram-negative strains tested, *i.e.* 1.5% of all submitted compounds. While this number is rather low, it is still significantly larger than the overall CO-ADD value of 0.16%, and is consistent with the inherent difficulty of finding compounds active against Gram-negative bacteria (*e.g.* a GSK screen of 500 000 k synthetic compounds against *S. aureus* and *E. coli* found thousands of Gram-positive actives with 300 considered for further evaluation, but no ‘non-nuisance’ Gram-negative hits^29^).

Gallium, palladium, silver, cadmium, iridium and platinum show the highest overall success rate. However, the overall numbers of submitted cadmium and gallium complexes were very low (5 and 6 respectively), limiting the ability to make general statements. Historically, both silver and gallium have been known to possess antimicrobial activity, a trend that is somewhat confirmed here.^30^–^34^ However, a significant portion of the silver complexes evaluated in this study also displayed cytotoxicity. While Pd, Ir and Pt complexes showed promising activities, these elements are relatively rare and expensive, possibly limiting their use as clinical antibiotics if large doses are required. Amongst the more common elements, iron stands out as one of the least effective metals: of 108 compounds tested, only 20 showed any kind of activity and *all* of those turned out to be cytotoxic and/or haemolytic, ruling out this subsample of iron complexes as possible antibiotics.

**Fig. 5:**
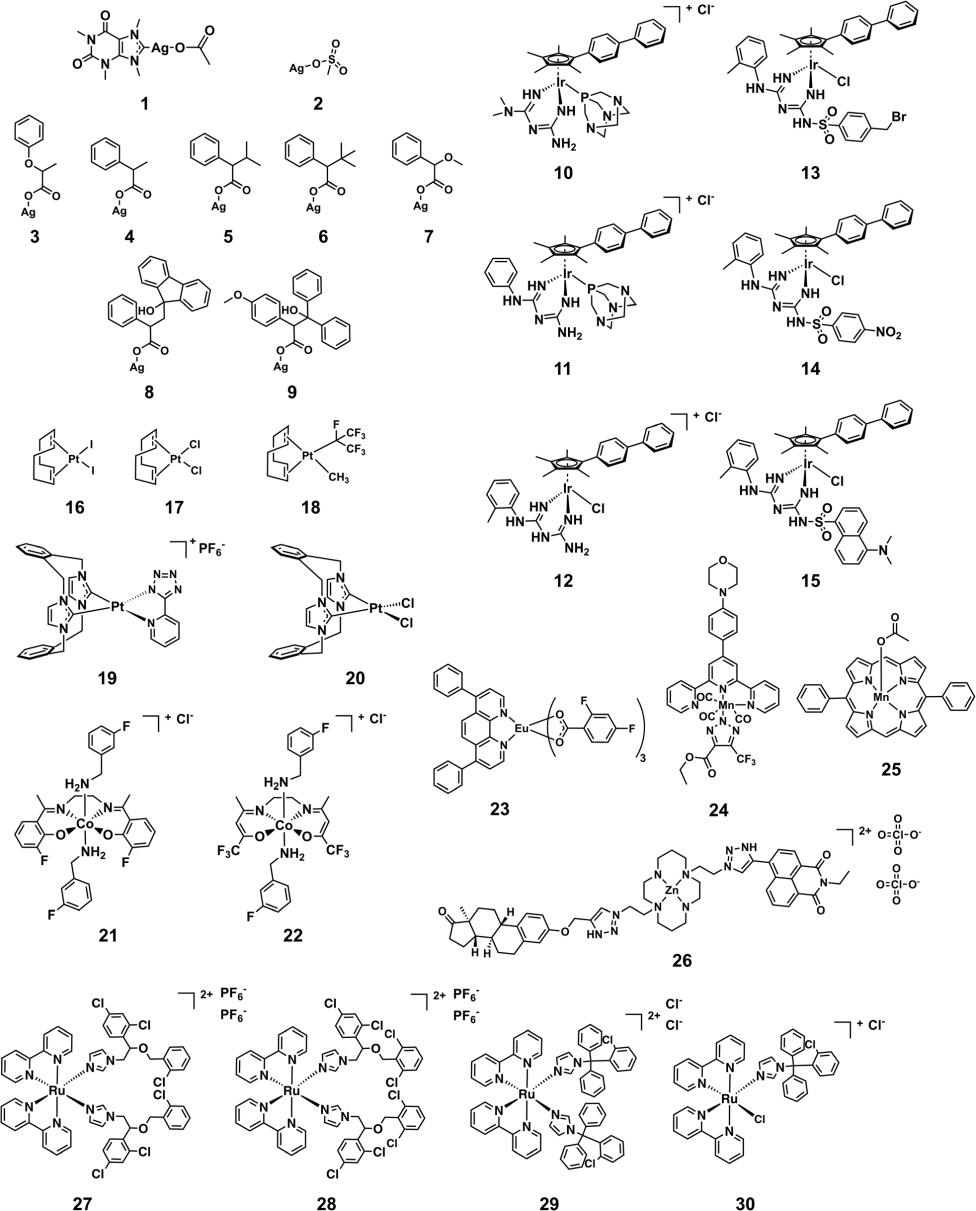
Structures of metal complexes that showed activity against strains and were not toxic and/or haemolytic to mammalian cells at the same concentration.

Fig. 5 shows the structures of the 30 compounds with activity against at least one bacterial strain and no toxicity or haemolytic properties at the highest tested concentration. In general, multiple examples of a submitted compound series are present, but in each case there are usually multiple similar compounds that are either not active or cytotoxic and/or haemolytic to human cells, providing some evidence of potential for further optimization and structure–activity relationship investigations. The structures of the exclusively antifungal compounds are not shown here and will be discussed elsewhere. The synthesis and characterization of all compounds shown here except silver complexes **2–9** has been previously reported in peer-reviewed publications (Table S3†). Since the exact structure of the silver carboxylates is not known, they are shown as carboxylate-coordinated species. In reality, these compounds are probably present as carboxylate-bridged oligomers.

An issue that is often raised for medicinal applications regarding metal complexes, but also organic molecules, is their stability in biological systems. While inertness is certainly not a requirement for good activity, as is showcased by cisplatin, it is paramount to understand the behavior of these compounds in biological systems. Stability studies in both common solvents as well as biological media and human blood should therefore be conducted for any metal-containing compound if it is to be considered for further development. In the case of the structures showcased in this work, a few general remarks can be made about their putative stability. The silver complexes **1–9** are likely to dissociate at some point when exposed to biological media. The different activity profiles of the compounds (*vide infra*) suggest that the nature of the ligand can dictate the time and place where the dissociation will occur, modulating the biological effect of the released silver ions. The other compounds are unlikely to dissociate to the point of releasing the metal ion, but some degree of ligand substitution is probable for most complexes highlighted here. The platinum compounds **16–18** and **19** are likely to undergo ligand exchange reactions when exposed to solvents and media. Reminiscent of cisplatin these reactions might actually lead to the formation of the active species in these cases. The axial ligands of **21** and **22** are known to be labile and are likely to undergo substitution reactions *in vitro* as well.^35^ Similarly, the chlorido and 1,3,5-triaza-7-phosphaadamantane (PTA) ligands in complexes **10–15** are prone to substitution.^36^ Ligand exchanges are conceivable for **23–25** but the biological behavior of these types of compounds has not been studied extensively yet. Ruthenium complex **27** has been shown to be stable in water for at least 24 h while the chloride ligand of **30** is readily replaced by water. However, the monodentate ligands have been reported to be released by light irradiation (520 nm, 1 h, 53 J cm^–2^) in the case of **27** and by inference this is probable for **28** and **29** as well, showcasing an example of interesting alternative modes of action that are possible with metal complexes.^37^ The fact that many of the more active compounds are likely to undergo ligand-exchange reactions when exposed to solvents and/or media suggests that the shown structures are potentially “prodrug-like” molecules and highlights that a traditional medicinal chemistry mindset cannot be applied indiscriminately to metal complexes.

Table 1 shows the MIC values of these 30 compounds against the CO-ADD panel of microorganisms as determined by broth microdilution assay. The xanthine derived *N*-heterocyclic carbene (NHC) complex of silver(i) (**1**) turned out to be the compound with best broad-spectrum activity, showing moderate MICs against all tested strains and no cytotoxicity and/or haemolysis.^38^ Compounds **2–9** contain Ag(i) with various carboxylate ligands. Interestingly, they show distinct activity profiles and several similar compounds did not show any activity, suggesting that different silver–ligand combinations lead to significantly different biological behavior, and that the silver content alone is not responsible for the observed activity. The antibacterial properties of some carboxylate silver(i) complexes have been summarized in a recent review article.^39^ NHC–silver carbenes are amongst the more intensely investigated metal complexes for antibacterial activity, and many more silver compounds have been reported, with the results summarized in several review articles.^40^–^42^ Furthermore, new advances have revealed detailed information on possible modes of action for silver-based antimicrobial compounds.^43^,^44^ Silver itself is known to be an antimicrobial agent, with varying activity depending on form (salts *vs.* nanoparticles *vs.* colloidal forms), so analysis of activity of silver complexes is complicated by this inherent activity.^45^,^46^ Altogether, our data support the published literature and highlights that silver compounds are a proven starting point for preparation of new potential antibiotic compounds.

**Table 1:** MIC and cytotoxicity values for the 30 metal complexes that showed activity against at least one bacterial strain. G–ve; Gram-negative bacteria, G+ve; Gram-positive bacteria, N.d; not determined

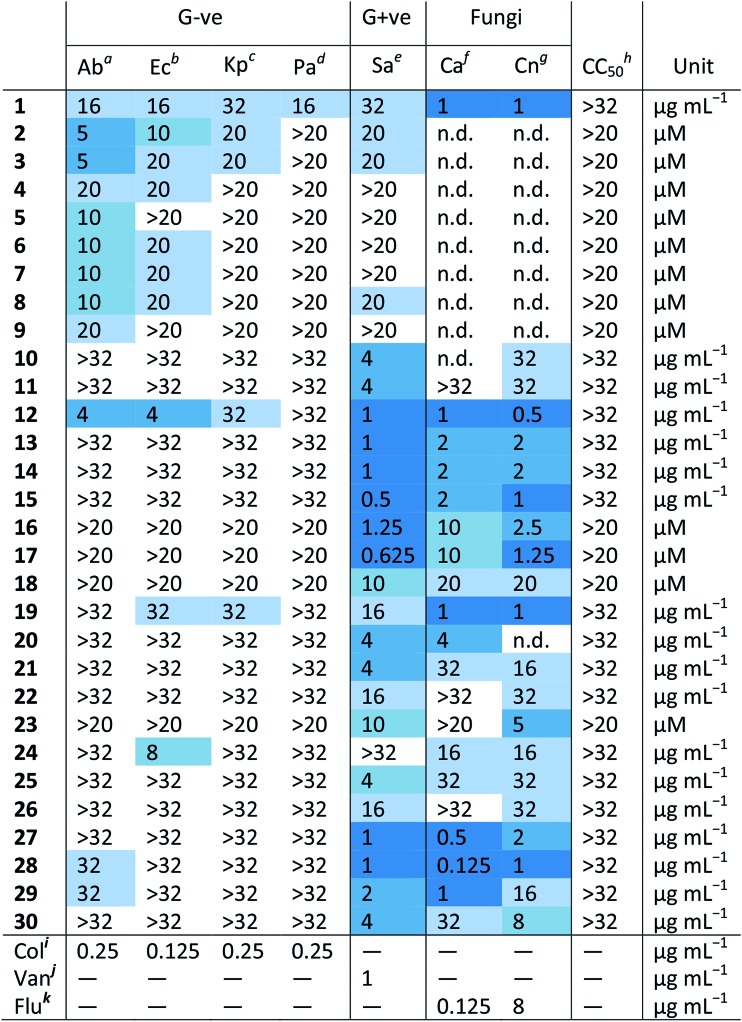

^*a*^Ab – *Acinetobacter baumannii* ATCC 19606.

^*b*^Ec – *Escherichia coli* ATCC 25922.

^*c*^Kp – *Klebsiella pneumoniae* ATCC 700603 (K6; ESBL SHV-18).

^*d*^Pa – *Pseudomonas aeruginosa* ATCC 27853.

^*e*^Sa – *Staphylococcus aureus* ATCC 43300 (MRSA).

^*f*^Ca – *Candida albicans* ATCC 90028.

^*g*^Cn – *Cryptococcus neoformans var grubii* H99 ATCC 208821.

^*h*^CC_50_ – HEK293 human embryonic kidney cells ATCC CRL-1573.

^*i*^Colistin.

^*j*^Vancomycin.

^*k*^Fluconazole.

Amongst the series of iridium(iii) complexes **10-15**, only **12** showed activity against some of the Gram-negative strains, in addition to the overall excellent activity of this compound series against the two fungal strains and MRSA. The group submitting these compounds have recently reported a detailed study on a larger series of iridium-based complexes, in which they describe potent activity against a series of Gram-negative, Gram-positive and fungal strains in conjunction with high complex stability and generally low toxicity. Additionally, synergistic activity with the antibiotic vancomycin was demonstrated, together with the ability to disrupt bacterial biofilms.^36^ By comparing the active structures with similar but inactive analogs, it becomes clear that the overall structure of the complex is crucial for the activity of these compounds. For example, analogous complexes with no phenyl or only one phenyl substituent on the cyclopentadiene ligand (**Ir-2** and **Ir-3**, ESI†), show almost no antimicrobial activity (MRSA, MIC = 32 and 16 μg mL^–1^, respectively, with no activity against the other organisms tested). The same is true for the biguanide ligand on its own (**Ir-1**, inactive at the highest tested concentration). Furthermore, small changes of the ligand, like the switch from a methyl-substituted to a fluoro-substituted phenyl group, or exchange of the chloro-ligand to a bromo or iodo, all produced compounds with elevated cytotoxicity but also broad activity against the other tested organisms (**Ir-4**, **Ir-5** and **Ir-6**, ESI†). Taken together this example illustrates that the observed activity does not derive solely from the metal ion or the ligand system, but that in this case the whole is more than the sum of its parts.

All the platinum complexes (**16–20**) were generally active against MRSA and the two tested fungal strains. The platinum-cyclooctadiene type complexes have been studied mainly as precursors, *e.g.* for metal organic vapor deposition (MOCVD) or supercritical fluid reactive deposition (SFRD) for the manufacture of optoelectronics or catalysts.^47^,^48^ In addition to structures to **17** and **18**, seven similar structures showed selective activity against the two fungal strains tested. Previously reported compounds of this class showed elevated toxicity against HeLa cells.^49^ Further studies to determine the active species of these compounds are certainly required. All these platinum compounds feature the metal in a +2 oxidation state. Recently, investigations into the application of Pt(iv) complexes as prodrugs that are reduced to Pt(ii) at the target site have gained a lot of attention.^9^ This principle could potentially also be applied for antibacterial purposes. Further investigations into these types of complexes certainly seem warranted. Compound **19** is the only platinum agent that displayed mild activity against *E. coli* and *K. pneumoniae*. Compounds **19** and **20** were previously reported for their luminescent properties.^50^ Two similar complexes (**Pt-1** and **Pt-2**, ESI†) were inactive.

The cobalt(iii) complexes **21** and **22** showed some activity against MRSA and the fungal strains. A detailed account on the physical properties and ligand substitution reactions of these complexes was recently published.^35^ These compounds also represent the only entries for this structure class so further studies will be needed to elucidate the full potential of these cobalt-based derivatives.

The four ruthenium(ii) complexes (**27–30**), showed excellent activity against MRSA as well as the two fungal strains. Two of them also showed mild activity against the Gram-negative strain *A. baumannii*. As mentioned earlier, compound **27** was reported previously for promising light-mediated activity against cancer cells.^37^ Possibly the activity of these compounds could be even better upon light irradiation. In general, ruthenium complexes, particularly polynuclear ones, have been reported to have favorable antimicrobial properties already in the 1950s by Dwyer^51^–^53^ and more recently by the groups of Collins and Keene.^54^–^60^ Together with very recent work on promising dinuclear ruthenium-based complexes, these results highlight that on top of their excellent anticancer activity, ruthenium-based compounds could also have potential antibacterial applications.^61^–^65^

Of the remaining entries, the manganese complex **24** stands out for its specific activity against *E. coli*, with the unrelated Mn compound **25** showing activity against only MRSA. Compound **25** has been studied previously as a superoxide dismutase and catalase mimic.^66^ The europium(iii) phenanthroline complex (**23**) and zinc(ii) cyclam complex (**26**) have unusual structures and their antibacterial activities are limited to MRSA strains. The europium complex is part of the lanthanide fluorobenzoate class of compounds.^67^,^68^ It has good luminescent properties which make it suitable for applications in cellular imaging, immunoassays and organic light-emitting diodes.^69^–^71^ The zinc complex **26** is related to a class of macrocyclic metal complexes recently shown to have promising activity against *Mycobacterium tuberculosis*,^72^,^73^ and was synthesized as part of a broader project to develop functionalized macrocycles for biological applications.^74^,^75^ The unusual structure of these compounds makes them interesting scaffolds for further studies into their biological behaviour.

There appeared to be some general species selectivity among the Gram-negative activity, with trends towards greater potency against *A. baumannii* and *E. coli* compared to *K. pneumoniae* and *P. aeruginosa*. However, given the limited number of active compounds and the narrow window between the active MICs and the highest concentrations tested, these observations are preliminary. We do note that a 2014 report on polynuclear ruthenium complexes found reduced *P. aeruginosa* susceptibility.^76^

## Conclusion

We have analyzed 906 metal-containing compounds for their antibacterial and antifungal activity and found an impressive success rate when compared to purely organic compounds. The syntheses of some of these compounds have previously been reported and the antibacterial properties of 7 of the 30 shown structures were partially described elsewhere. The 23 remaining complexes have not been reported for their antimicrobial activity before. Our analysis of this set of compounds highlights the vast diversity of metals, types of ligands, and geometries that are possible with metal compounds. This finding is in good agreement with the recent report by Morrison *et al.* demonstrating the utility of metal complexes in accessing underexplored chemical space for drug development.^26^ It is evident that more compounds need to be tested before detailed trends can be determined and ultimately predicted. Nevertheless, these structures may serve as starting points for further structure–activity explorations as they constitute interesting and much needed new structural classes with antimicrobial activity that could potentially be improved upon. It is possible that for many compounds, the lack of Gram-negative activity is due to limited cellular entry or susceptibility to extensive efflux pump removal from the bacteria, and additional structural modifications might be able to overcome these barriers. This work underscores the far-reaching diversity and potential that metal compounds can bring to the field of antibiotic drug discovery. An analysis of the active but non-toxic hits provides some preliminary insights into the more suitable elements and structures while also pointing out where the chemical space remains uncharted. There are obviously substantial barriers that must be overcome that are not addressed in this study, particularly *in vivo* stability and toxicity. Another question that will have to be addressed is the mechanism of action of these compounds, and indeed what the active compound is, *e.g.* what interactions occur within the bacteria that may modify the initial metal complex before the compound kills the cells. Several general modes of action can be envisioned, and these will likely vary between different classes of compounds. A metal complex can be inert, *i.e.* the ligand framework stays intact and the whole compound binds a specific bacterial target. The compound can be partially labile, *i.e.* some ligands could be exchanged in different environments and generate a species that can bind a bacterial target or is by itself reactive/toxic. It can also be the released ligands that are responsible for the observed activity. Finally, the activity could be fully metal-mediated and the ligand framework merely serves as a vehicle to deliver the active metal ion. Previously, Alessio and coworkers have categorized anticancer metal complexes based on their possible mode of action, a classification that can be applied to metal-based antibiotics as well.^28^ The group of Sun has very recently reported a series of detailed studies on the targets and modes of action of gallium, silver and bismuth against bacteria.^43^,^77^–^79^ Such metalloproteomic approaches will be very valuable to further determine the behavior and targets of these compounds and aid the design of improved metalloantibiotics.

The combination of an extended arsenal of possible modes of action with a broader coverage of three-dimensional chemical space make a strong case for metal complexes as potential (antibiotic) drug candidates with some key advantages over their organic counterparts.

Given the scarcity of new antibiotics currently in the pipeline, we hope to raise the awareness of the potential of metal compounds for antimicrobial applications and inspire further investigations into their development. In particular, we wish to encourage additional researchers to send their metal compounds to CO-ADD so that we can expand this open database and boost investigations into metal compounds as potential antibiotics.

## Methods

### Purity of compounds

All compounds were obtained as dried powders from collaborators and confirmed by the collaborator to be at >95% purity. No further purification was performed by CO-ADD. The dry compounds were dissolved to a concentration of 10 mg mL^–1^ or 10 mM in DMSO. Samples were diluted to a final testing concentration of 32 μg mL^–1^ or 20 μM, depending on the available stock solution, while keeping the final DMSO concentration to a maximum of 0.5%, and serially diluted 1 : 2 fold for 8 times.

### Antibacterial assays

For the all the bacterial assays, each strain was cultured in Cation-adjusted Mueller Hinton broth (CAMHB; Bacto Laboratories 212322) at 37 °C overnight. A sample of each culture was then diluted 40-fold in fresh CAMHB and incubated at 37 °C for 1.5–3 h. The resultant mid-log phase cultures were diluted with CAMHB (CFU mL^–1^ measured by OD_600_), then added to each well of the compound-containing plates (384-well non-binding surface (NBS) plates; Corning CLS3640), giving a cell density of 5 × 10^5^ CFU mL^–1^ and a total volume of 50 μL. Plates were covered and incubated at 37 °C for 18 h without shaking. Inhibition of bacterial growth was determined measuring absorbance at 600 nm (OD_600_), using media only as negative control and bacteria without inhibitors as positive control. MIC values were determined as the lowest concentration at which the growth was inhibited at ≥80%. Colistin sulfate (Sigma C4461) and vancomycin HCl (Sigma 861987) were used as internal controls on each plate for Gram-negative and Gram-positive bacteria, respectively.

### Antifungal assays

For the fungal assays, both fungi (yeast) strains were cultured for 3 days on Yeast Extract-Peptone Dextrose (YPD; Becton Dickinson 242720) agar at 30 °C. A yeast suspension of 1 × 10^6^ to 5 × 10^6^ CFU mL^–1^ (as determined by OD_530_) was prepared from five colonies from the agar plates, and subsequently diluted with Yeast Nitrogen Base media (YNB; Becton Dickinson 233520), and added to each well of the compound-containing plates (384-well plates, NBS; Corning CLS3640) giving a final cell density of 2.5 × 10^3^ CFU mL^–1^ and a total volume of 50 μL. Plates were covered and incubated at 35 °C for 36 h without shaking. Growth inhibition of *C. albicans* was determined by measuring absorbance at 630 nm (OD_630_), while the growth inhibition of *C. neoformans* was determined by measuring the difference in absorbance between 600 and 570 nm (OD_600–570_), after the addition of resazurin (0.001% final concentration; Sigma R7017) and incubation at 35 °C for 2 h, using media-only as negative control and fungi without inhibitors as positive control. MIC values were determined as the lowest concentration at which the growth was inhibited at ≥80%. Fluconazole (Sigma F8929) was used as internal control on each plate for both strains.

### Cytotoxicity assays

HEK293 ATCC CRL-1573 human embryonic kidney cells were counted manually in a Neubauer haemocytometer and added to compound-containing plates (384-well plates, tissue culture treated (TC); Corning CLS3712) giving a final density of 5000 cells per well in a and a total volume of 50 μL, using Dulbecco's Modified Eagle Medium (DMEM; Life Technologies 11995-073) with 10% Foetal Bovine Serum (FBS; GE SH30084.03). The cells were incubated together with the compounds for 20 h at 37 °C in 5% CO_2_. Cytotoxicity (or cell viability) was measured by fluorescence, ex: 560/10 nm, em: 590/10 nm (F560/590), after addition of 5 μL of 25 μg mL^–1^ resazurin (2.3 μg mL^–1^ final concentration; Sigma R7017) and after further incubation for 3 h at 37 °C in 5% CO_2_, using media-only as negative control and cells without inhibitors as positive control. CC_50_ (concentration at 50% cytotoxicity) were calculated by curve-fitting the inhibition values *vs.* log(concentration) using a sigmoidal dose–response function, with variable fitting values for bottom, top and slope. Tamoxifen (Sigma T5648) was used as internal control on each plate.

### Haemolysis assays

Human whole blood (Australian Red Cross) was washed three times with 3 volumes of 0.9% NaCl and resuspended in a concentration of 0.5 × 10^8^ cells per mL, determined by manual cell count in a Neubauer haemocytometer. Washed cells were added to compound containing plates (384-well polypropylene plates (PP); Corning 3657) for a final volume of 50 μL, shaken and incubated for 1 h at 37 °C. After incubation, the plates were centrifuged at 1000 g for 10 min to pellet cells and debris, 25 μL of the supernatant was then transferred to reading plates (384 well, polystyrene plated (PS), Corning CLS3680), with haemolysis determined by measuring the supernatant absorbance at 405 mm (OD405), using cells without inhibitors as negative control and cells with 1% Triton X-100 (Sigma T8787) as positive control. HC_10_ and HC_50_ (concentration at 10% and 50% haemolysis, respectively) were calculated by curve fitting the inhibition values *vs.* log(concentration) using a sigmoidal dose–response function with variable fitting values for top, bottom and slope. Melittin (Sigma M2272) was used as internal control on each plate.

## Author contributions

A. F conceived the project concept to analyse CO-ADD data for metal complexes. A. F. and J. Z. analysed the data and generated the graphics. M. A. T. B., M. A. C., J. Z., and A. G. E. founded the screening initiative CO-ADD and collected the microbiological data. A. F., J. Z. and M. A. T. B. composed the manuscript. M. A. C. and A. G. E. provided important insights and feedback on the manuscript. M. B., S. B., C. B., F. C., C. D., G. D., N. J., A. P. K., A. M. M., M. M., J. M., H. A. M., A. K. R., P. J. R., P. J. S., M. H. T., C. E. W., and J. J. W. prepared, characterized and submitted the compounds highlighted in this work for antimicrobial activity. All authors discussed, commented and approved the final manuscript.

## Conflicts of interest

There are no conflicts to declare.

## Supplementary Material

Supplementary information
