# Correction: Metal complexes as a promising source for new antibiotics

**DOI:** 10.1039/d0sc90075c

**Published:** 2020-04-16

**Authors:** Angelo Frei, Johannes Zuegg, Alysha G. Elliott, Murray Baker, Stefan Braese, Christopher Brown, Feng Chen, Christopher G. Dowson, Gilles Dujardin, Nicole Jung, A. Paden King, Ahmed M. Mansour, Massimiliano Massi, John Moat, Heba A. Mohamed, Anna K. Renfrew, Peter J. Rutledge, Peter J. Sadler, Matthew H. Todd, Charlotte E. Willans, Justin J. Wilson, Matthew A. Cooper, Mark A. T. Blaskovich

**Affiliations:** Centre for Superbug Solutions, Institute for Molecular Bioscience, The University of Queensland St. Lucia Queensland 4072 Australia angelo.frei.ch@gmail.com m.blaskovich@uq.edu.au; School of Molecular Sciences, The University of Western Australia Stirling Highway 6009 Perth Australia; Institute of Organic Chemistry, Karlsruhe Institute of Technology (KIT) Fritz-Haber-Weg 6 76131 Karlsruhe Germany; Institute of Biological and Chemical Systems – Functional Molecular Systems (IBCS-FMS), Karlsruhe Institute of Technology (KIT) Hermann-von-Helmholtz-Platz 1 D-76344 Eggenstein-Leopoldshafen Germany; School of Medical Sciences (Discipline of Pharmacology), University of Sydney Australia; Department of Chemistry, University of Warwick Gibbet Hill Road Coventry CV4 7AL UK; Antimicrobial Screening Facility, School of Life Sciences, University of Warwick Gibbet Hill Road Coventry CV4 7AL UK; Institute of Molecules and Matter of Le Mans (IMMM), Le Mans Université UMR 6283 CNRS France; Department of Chemistry and Chemical Biology, Cornell University Ithaca NY 14853 USA; Chemistry Department, Faculty of Science, Cairo University Egypt; School of Molecular and Life Sciences – Curtin Institute for Functional Materials and Interfaces, Curtin University Kent Street 6102 Bentley WA Australia; School of Chemistry, University of Leeds Woodhouse Lane Leeds LS2 9JT UK; School of Chemistry, The University of Sydney Sydney NSW 2006 Australia; School of Pharmacy, University College London London WC1N 1AX UK

## Abstract

Correction for ‘Metal complexes as a promising source for new antibiotics’ by Angelo Frei *et al.*, *Chem. Sci.*, 2020, **11**, 2627–2639.

In the original manuscript, the affiliation for author Peter J. Rutledge was incorrectly labelled as ‘*e*’, and should have been labelled as affiliation ‘*m*’ – School of Chemistry, The University of Sydney, Sydney, NSW 2006, Australia. Please see the above author list for the corrected affiliation.

The Royal Society of Chemistry apologises for these errors and any consequent inconvenience to authors and readers.

