# Supplementary material for: Metal complexes as a promising source for new antibiotics
Source: Chem Sci. 2020 Feb 12;11(10):2627–39. doi: 10.1039/c9sc06460e (PMC7069370; doi:10.1039/c9sc06460e)
Supplement: Supplementary file 1 [file SC-011-C9SC06460E-s001.pdf]

Electronic Supplementary Material (ESI) for Chemical Science.
This journal is © The Royal Society of Chemistry 2020

Metal Complexes as a Promising Source for New Antibiotics

Angelo Frei[a†], Johannes Zuegg[a†], Alysha G. Eliott[a], Murray Baker[b], Stefan Braese[c], Christopher Brown[d], Feng Chen[f], Christopher Dowson[e], Gilles Dujardin[g], Nicole Jung[c], A. Paden King[h], Ahmed M. Mansour[i], Massimiliano Massi[j], John Moat[e], Heba A. Mohamed[i], Anna K. Renfrew[l], Peter. J. Rutledge[d], Peter J. Sadler[f], Matthew H. Todd[l,m], Charlotte E. Willans[j], Justin J. Wilson[h], Matthew A. Cooper[a], and Mark A. T. Blaskovich[a]

[a]Institute for Molecular Bioscience, The University of Queensland, St. Lucia, Queensland 4072 (Australia).

[b]School of Molecular Sciences, The University of Western Australia, Stirling Highway, 6009 Perth (Australia).

[c]Institute of Organic Chemistry, Karlsruhe Institute of Technology, Fritz-Haber-Weg 6, 76131 Karlsruhe (Germany). Institute of Toxicology and Genetics, Karlsruhe Institute of Technology, Hermann-von-Helmholtz-Platz 1, 76344 Eggenstein-Leopoldshafen (Germany).

[d]School of Medical Sciences (Discipline of Pharmacology), University of Sydney (Australia).

[e]School of Life Sciences and [f]Department of Chemistry, University of Warwick, Gibbet Hill Road, Coventry CV4 7AL (UK).

[g]Institute of Molecules and Matter of Le Mans (IMMM), UMR 6283 CNRS, Le Mans Université (France).

[h]Department of Chemistry and Chemical Biology, Cornell University, Ithaca, NY 14853 (USA).

[i]Chemistry Department, Faculty of Science, Cairo University (Egypt).

[j]School of Molecular and Life Sciences – Curtin Institute for Functional Materials and Interfaces, Curtin University, Kent Street, 6102 Bentley WA (Australia).

[k]School of Chemistry, University of Leeds, Woodhouse Lane, Leeds LS2 9JT (UK).

[l]School of Chemistry, The University of Sydney, Sydney, NSW 2006 (Australia).

[m]U.K. School of Pharmacy, University College London, London, WC1N 1AX (UK).

[†]*joint first authors*

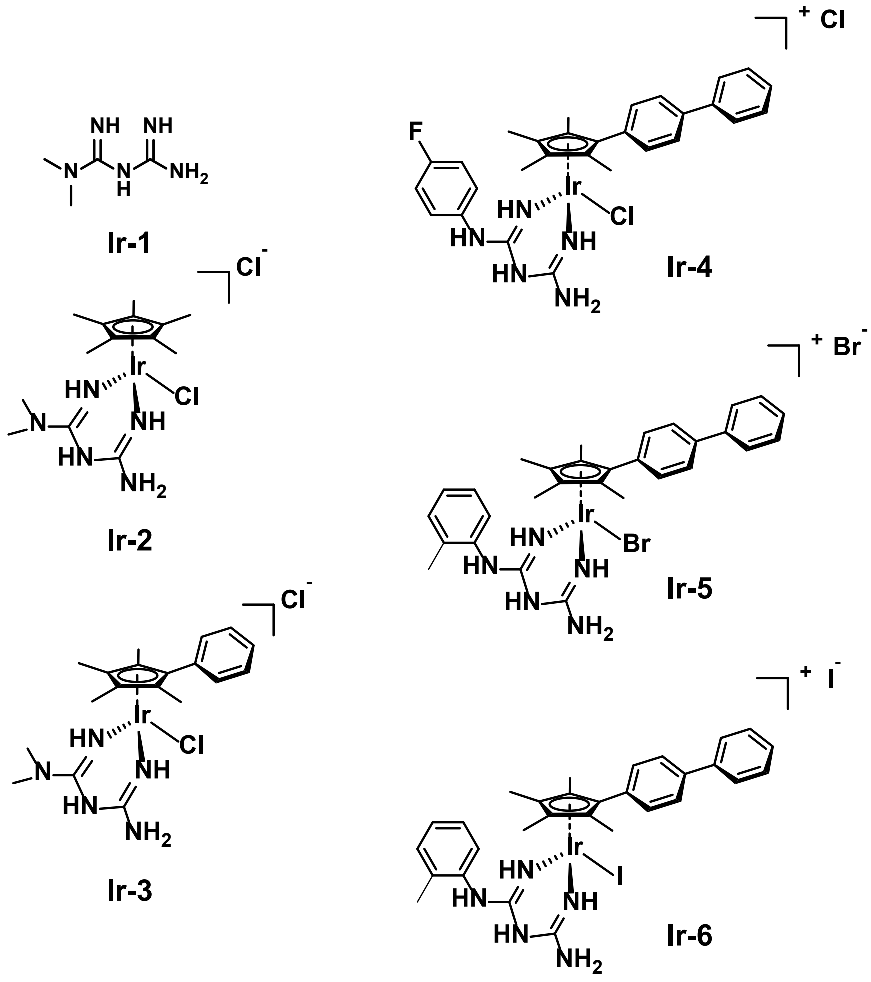

	G-ve				G+ve	Fungi			
	Ab[a]	Ec[b]	Kp[c]	Pa[d]	Sa[e]	Ca[f]	Cn[g]	CC_{50}[h]	
**Ir-1**	>32	>32	>32	>32	>32	>32	>32	>32	µg/ml
**Ir-2**	>32	>32	>32	>32	32	>32	>32	>32	µg/ml
**Ir-3**	>32	>32	>32	>32	16	>32	>32	28.1	µg/ml
**Ir-4**	4	8	>32	>32	1	1	0.5	19.3	µg/ml
**Ir-5**	4	8	8	>32	1	1	0.5	23.7	µg/ml
**Ir-6**	16	8	16	>32	1	1	0.5	17.0	µg/ml

[a]Ab - *Acinetobacter baumannii* ATCC 19606, [b]Ec - *Escherichia coli* ATCC 25922; [c]Kp - *Klebsiella pneumoniae* ATCC 700603, [d]Pa - *Pseudomonas aeruginosa* ATCC 27853, [e]Sa - *Staphylococcus aureus* ATCC 43300, [f]Ca - *Candida albicans* ATCC 90028, [g]Cn - *Cryptococcus neoformans* ATCC 208821, [h]CC_{50} - HEK293 cells

Figure S1. Structures and antimicrobial activity for related active and inactive iridium-based complexes similar to **10-15**.

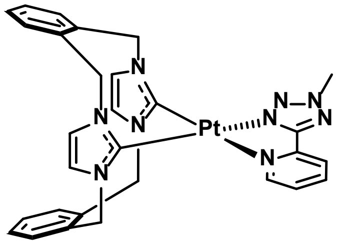

Pt-1

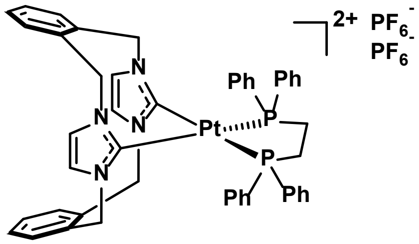

Pt-2

	G-ve				G+ve	Fungi			
	Ab[a]	Ec[b]	Kp[c]	Pa[d]	Sa[e]	Ca[f]	Cn[g]	CC_{50}[h]	
**Pt-1**	>32	>32	>32	>32	>32	32	32	>32	μg/ml
**Pt-2**	>32	>32	>32	>32	32	>32	32	>32	μg/ml

[a]Ab - *Acinetobacter baumannii* ATCC 19606, [b]Ec - *Escherichia coli* ATCC 25922; [c]Kp - *Klebsiella pneumoniae* ATCC 700603, [d]Pa - *Pseudomonas aeruginosa* ATCC 27853, [e]Sa - *Staphylococcus aureus* ATCC 43300, [f]Ca - *Candida albicans* ATCC 90028, [g]Cn - *Cryptococcus neoformans* ATCC 208821, [h]CC_{50} - HEK293 cells

Figure S2. Structures and antimicrobial activity for cluster of related inactive platinum-based complexes similar to **19-20**.

Characterization Data for tested compounds

Compound	X-ray Crystal Structure	Synthesis and Characterization Published
**1**	Structure for analogous compounds available[1]	Yes[1]
**2-9**	No	No
**10-15**	Structures for analogous compounds available[2]	Yes[2]
**16 -18**	Structure for analogous compounds available[3]	Yes[3]
**19-20**	No	Yes[4]
**21-22**	Yes[5]	Yes[5]
**23**	Yes[6]	Yes[6]
**24**	No	*Submitted*
**25**	No	Yes[7]
**26**	No	Yes[8]
**27-30**	No	Compound **27**[9]

References

[1] H. A. Mohamed, B. R. M. Lake, T. Laing, R. M. Phillips, C. E. Willans, *Dalton Trans.* **2015**, *44*, 7563-7569.

[2] F. Chen, J. Moat, D. McFeely, G. Clarkson, I. J. Hands-Portman, J. P. Furner-Pardoe, F. Harrison, C. G. Dowson, P. J. Sadler, *J. Med. Chem.* **2018**, *61*, 7330-7344.

[3] M. Enders, B. Görling, A. B. Braun, J. E. Seltenreich, L. F. Reichenbach, K. Rissanen, M. Nieger, B. Luy, U. Schepers, S. Bräse, *Organometallics* **2014**, *33*, 4027-4034.

[4] K. D. M. MaGee, P. J. Wright, S. Muzzioli, C. M. Siedlovskas, P. Raiteri, M. V. Baker, D. H. Brown, S. Stagni, M. Massi, *Dalton Trans.* **2013**, *42*, 4233-4236.

[5] A. P. King, H. A. Gellineau, S. N. MacMillan, J. J. Wilson, *Dalton Trans.* **2019**, *48*, 5987-6002.

[6] A. S. Kalyakina, V. V. Utochnikova, I. S. Bushmarinov, I. V. Ananyev, I. L. Eremenko, D. Volz, F. Rönicke, U. Schepers, R. Van Deun, A. L. Trigub, Y. V. Zubavichus, N. P. Kuzmina, S. Bräse, *Chem. – Eur. J.* **2015**, *21*, 17921-17932.

[7] R. A. Rosenthal, K. D. Huffman, L. W. Fisette, C. A. Damphousse, W. B. Callaway, B. Malfroy, S. R. Doctrow, *J. Biol. Inorg. Chem.* **2009**, *14*, 979-991.

[8] M. Spain, J. K. H. Wong, G. Nagalingam, J. M. Batten, E. Hortle, S. H. Oehlers, X. F. Jiang, H. E. Murage, J. T. Orford, P. Crisologo, J. A. Triccas, P. J. Rutledge, M. H. Todd, *J. Med. Chem.* **2018**, *61*, 3595-3608.

[9] N. Karaoun, A. K. Renfrew, *Chem. Comm.* **2015**, *51*, 14038-14041.
